# Assessment of novel electrophysiology simulator—a survey study

**DOI:** 10.1186/s41077-024-00280-9

**Published:** 2024-02-14

**Authors:** Maciej Koźlik, Jędrzej Kosiuk, Michał Cogiel, Jan Kost, Daria Hemmerling, Michał Staniszewski, Agnieszka Szczęsna, Wojciech Wojakowski, Tomasz Jadczyk

**Affiliations:** 1 Division of Cardiology and Structural Heart Disease, Medical University of Silesia, Katowice, 40055 Poland; 2 Departament of Cardiology, Helios Klinik Koethen, Koethen, 06366 Germany; 3 QSystems.pro sp. z o.o, Mochnackiego 34, Bytom, 41907 Poland; 4 AGH University of Science and Technology, Kraków, 30059 Poland; 5 Department of Computer Graphics, Vision and Digital Systems, Silesian University of Technology, Akademicka 16, Gliwice, 44100 Poland

**Keywords:** Simulator, Training, Electrophysiology, Questionnaire, Curriculum

## Abstract

**Background:**

Invasive electrophysiology (EP) training requires intellectual skills related to the interpretation of intracardiac electrograms. The classic approach to the education of young electrophysiologists focused solely on theoretical knowledge and overseen procedures in patients as no real-life-like simulation of EP studies was available.

**Objective:**

The purpose of this study was to assess a novel tool for EP training based on fully interactive, online simulator providing real clinical experience to the users.

**Methods:**

EP simulator users access a system with simulated electrocardiogram, mimicking signals recorded by a catheter. Assessment of EP simulator by 40 electrophysiologists from 16 countries was collected via online questionnaire.

**Results:**

The realism of ECG signals was described as excellent or very good by 90% of responders, of intracardial signals by 82.5%. Realism of signal interactions and user experience was judged as excellent or very good by 75% and 70% accordingly. One hundred percent of users agree definitely or mostly that EP Simulator helps to translate theoretical into practical knowledge. Of responders, 97.5% would include it in EP training programs as it is extremely or very useful for training purposes in the opinion of 87.5%. Of responders, 72.5% think that training on EP simulator can potentially reduce the rate of complications. In 87.5%, the overall experience was completely or mostly satisfying and would be recommended by 100% of responders.

**Conclusion:**

EP simulator is a feasible tool for training of young electrophysiologist, and it may be potentially included in the cardiologist curriculum. We should particularly emphasize the positive respondents’ assessment of EP simulator overall realism.

**Supplementary Information:**

The online version contains supplementary material available at 10.1186/s41077-024-00280-9.

## Introduction

Invasive electrophysiology (EP) includes procedures responsible for the diagnosis and treatment of cardiac arrhythmias: analysis of the rhythm disorder mechanism, locating arrhythmia site (cardiac mapping), and treatment via catheter-based ablation techniques. EP requires a challenging training process. It is equally demanding in relation to both manual skills as well as intellectual abilities in interpretating intracardiac electrograms. This translates in a long learning curve [1]. Junior cardiologist has to obtain necessary knowledge in the field of diagnosing clinically important conditions with the use of electrocardiography (ECG) in order to use it in practice [2]. With the use of electrocardiographic signals, they have to locate the source of arrythmia in the heart. Moreover, they need to be trained in cardiac catheterization which means operating a small, flexible tube (a catheter) in the heart. According to the EHRA study from September 2023, the minimum duration of a strictly EP training should last for at least 2 years. In the past, there were no real-life EP simulators, so the whole education focused mostly on theoretical knowledge and overseen procedures. With the more universal access to internet connection and technology, the way of teaching evolves as well. Simulation method is useful for improving essential skills with minimal risk and friendly environment in the fields where a potential mistake can cause serious complications for a patient [3]. It is also useful for advanced operators to revise rarer conditions [4]. There are some studies which clearly show that simulation method of education can improve operator performance in EP [1]. We must remember that nowadays, technology develops rapidly which causes a constant compulsion to acquire new skills [3], and demand for simulation-learning methods will probably increase in the future [5]. When creating an EP simulator, the following criteria must be included: an EP simulator should be realistic, provide optimal training environment, present actual clinical cases, have accessible interface for a user, and include some additional tools or functions to make the training as efficient as possible.

The aim of this study was to assess a novel EP training tool (https://www.ep-simulator.com) which was designed to fulfil above mentioned criteria and provide users with real clinical experience.

EP simulator is a free and online simulator. In order to use it, the user needs a web browser on a device (for instance a computer or mobile phone), internet connection, and signing up.

## Methods

### Study design

EP simulator replicates EP recording system routinely used in cardiac electrophysiology laboratories and therefore enables easier transition from training to clinical situation. Users are able to access a fully functional system with simulated surface electrocardiogram and intracardiac electrograms mimicking signals recorded by a catheter located at high right atrium, His bundle, coronary sinus, and right ventricular apex. Normal electrophysiology study and the following cardiac arrhythmias are available for simulation: sick sinus syndrome, AV conduction blocks, atrial ectopy, atrial flutter and fibrillation, atrioventricular, and atrioventricular nodal reentrant tachycardia and ventricular tachycardias. Assessment by 40 electrophysiologists (mean experience 8 ± 6 years) from 16 countries was collected via online questionnaire.

In this study, we prepared a survey on Google Forms containing 13 questions. Every question provided 5 possible answers (answers were arranged gradually, from most positive answer to the most negative answer). For full transparency, we have added appendix information providing complete survey questions (Additional file 1).

**Fig. 1:**
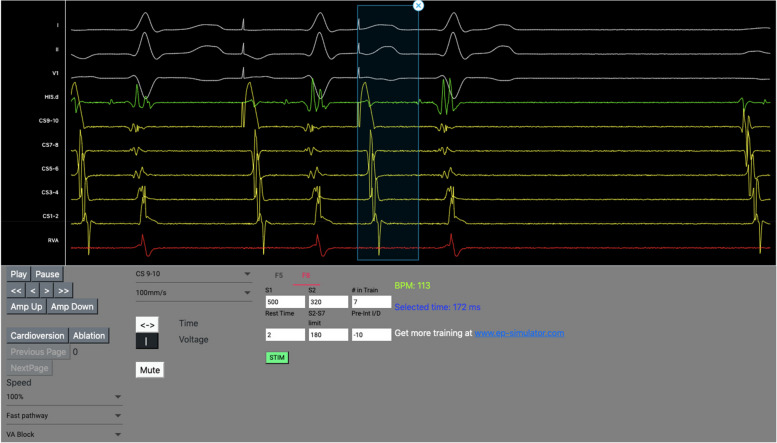
Example EP simulator interface

In order to simplify data presentation and statistical analysis, answers were merged: two most positive answers together, middle answer remained separate as neutral, and two most negative answers together. The questionnaire, with an access to EP simulator, was sent to practicing electrophysiologists with different levels of experience for systemic assessment. It was sent with an access to EP simulator to independent experts from European countries who willingly and voluntarily assessed EP simulator. The survey was launched in December 2022 and was closed at the beginning of January 2023. An image of intracardial signalization from EP online simulator available for users is presented in Fig. 1.

### Statistical analysis

Continuous variables were expressed as mean and standard deviation, and categorical variables were presented as numbers and percentages. For simplification of data presentation, answers were merged into positive answer, neutral answer, and negative answer groups to make 3 categories. All responses in detail and exact number of responses are presented in the appendix (Additional file 1). In order to explore possible differences in the assessment of the simulator within the cohort, the group was divided by mean age and experience level described as years in EP. Comparisons between groups were performed using *χ*^2^ test for categorical variables. Statistical analysis was performed using SPSS 29.0 for Mac OS. Values of *P* < 0.05 (two-tailed) were considered statistically significant.

## Results

### Respondents’ characteristics

Responses from 40 operators (75% men) with mean age 39 ± 6 and mean clinical experience of 8 ± 6 were collected. Respondents originated from 16 different countries: Germany (11), Poland (8), Austria (6), Romania (2), UK (2), Belgium (1), Croatia (1), Czech Republic (1), France (1), Greece (1), Hungary (1), Italy (1), Russia (1), Slovenia (1), Spain (1), and Turkey (1).

### Realism of electrophysiological simulations

Realism of ECG signals was assessed as excellent or very good by 90% of respondents. Of surveyed operators, 82.5% assessed realism of intracardial EGM signals as excellent or very good. For 75% of operators, realism of signal interactions was excellent or very good. Moreover, 70% of respondents assessed the realism of operators’ experience as excellent or very good.

### Optimal training environment and simulator usefulness assessment

**Fig. 2:**
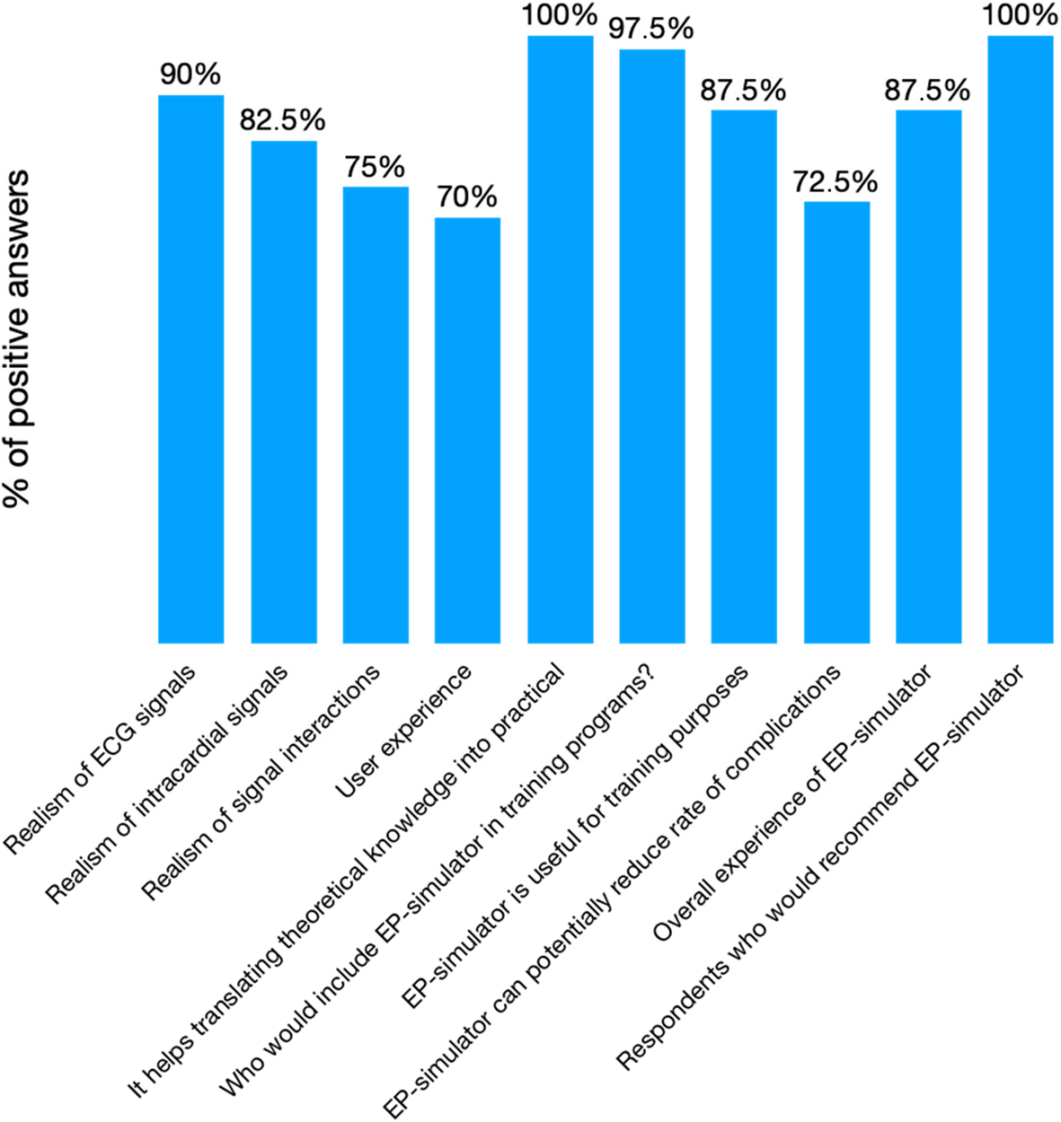
Percentage of positive answers (online questionnaire) assessing EP simulator

EP simulator was assessed as extremely or very useful for training purposes by 87.5% of operators and there were no negative answers (Fig. 2). Of surveyed respondents, 97.5% admitted that using EP simulator should be included in EP training programs and there were no negative answers. For 100% of respondents, it was definitely true or mostly true that EP simulator was a helpful tool in translating theoretical knowledge into practical skills.

Of operators, 87.5% were completely or mostly satisfied with the overall experience while using EP simulator and there were no negative answers. For 72.5% of surveyed respondents, EP simulator could potentially reduce procedural risk for patients. Last but not least, EP simulator would be recommended to a friend or colleague by 100% of respondents.

#### Scenarios in EP simulator

Scenarios available for simulation in the EP simulator were completely or mostly satisfying for 77.5% respondents. There were no negative answers in that respect.

#### User interface in EP simulator

For 85% of respondents, the EP simulator was completely or mostly intuitive and there were no negative answers.

#### Additional tools and functions in EP simulator

Of surveyed operators, 82.5% were completely or mostly satisfied with the additional functions of the EP simulator.

### Stratification of respondents’ assessment according to age and experience level

We divided respondents into two groups divided by mean age (39) and level of expertise (8 years). Younger group consisted of 18 operators and older of 22 operators. Twenty operators were classified as less experienced and 20 had higher level of expertise. There were no statistically significant differences between the groups regarding questions in any category.

## Discussion

In this study, we assessed for the first time an online training with a unique novel tool, i.e., EP simulator, and present study’s main findings. The vast majority of responses were highly positive and indicate both technical feasibility and great need for the availability of such programs in the environment of both more or less experienced electrophysiologists. Regardless of the negative answers, all respondents recommended EP simulator (40). So far, there are no simulators officially included in the training program of a young electrophysiologist.

ESC curriculum for interventional cardiology recommends the future use of simulation as a vital assessment tool. However, those educational resources are of limited availability due to scarcity and high costs [6]. Because of these limitations, AHA and ACC guidelines do not even discuss recommendation including such techniques in interventional cardiologist training guidelines [7].

Although the utilization of simulation for training purposes indisputably improves outcome and reduces time needed to reach acceptable level of expertise [8–10], there are no scalable and easily accessible training simulators for electrophysiologists [11].

In contrast, the current trend in medicine towards more sophisticated and technologically advanced treatment requires life-like and hands-on training. Furthermore, the increasing complexity of procedures extends the training duration as well as demands the availability of a large sample of training cases. This translates into an urgent need for simulated-based training. Thus, vast majority of our responders would include it in EP training programs and see it as an extremely useful for educational purposes. This universal agreement among responders regarding the value of the platform might be caused by achieved high realism of ECG signals, intracardial signals, and signal interactions. This fact clearly demonstrates technical possibilities of such online tools in regard to reflecting clinical reality. Giving a high fidelity of signals, EP simulator might be successfully used also for demonstration and teaching purposes.

The high realism of operator experience enables better preparation of young doctors for stressful and potentially harmful situations. Hence, training on EP simulator can potentially reduce the rate of complications and minimalize patient risk as expected by large majority of experts. Similar conclusions concerning medical simulation training in general appeared also in some other studies [12, 13]. Moreover, it can be speculated that more training in stress-free, comfortable, and friendly environment will have positive effect on operators.

What must be stressed is that EP simulator was recommended by experts as a valuable tool regardless of experience level and age. Responses of operators across all groups were homogenous—100% agreed that EP simulator helps to translate theoretical knowledge into practical knowledge, and all of them would recommend it to others.

The encouraging results of our study require further systematic assessment of the training progress via EP simulator.

We would like to emphasize the fact that EP simulator is a free and online simulator which can possibly be accessed from every place with internet connection, browser, and computer or mobile. It is a very economically effective way of transmitting knowledge and can be especially useful in low-income and developing countries.

## Limitation of this study

This study has typical limitations of a survey research attributed to target respondents and questionnaire design. The survey included a limited number of selected physicians (40), who originated only from developed countries. Participation was voluntary, and therefore, it was prone to selection bias. The questionnaire was sent to participants from a single source which is also a limitation and some under or overreporting cannot be excluded.

## Conclusion

EP simulator is a fantastic solution for young electrophysiologist, and it can be potentially included in cardiologist training program. The positive respondents’ assessment of EP simulator overall realism should be especially emphasized. Regardless of age and level of experience, operators are in favor of EP simulator and recommend it for training purposes.

### Supplementary Information

**Additional file 1.**

## Data Availability

The data underlying this article will be shared on reasonable request to the corresponding author.

## Abbreviations

AV: Atrioventricular
ECG: Electrocardiography
EGM: Electrogram
EP: Electrophysiology
