# Supplementary figures and images for: Assessment of novel electrophysiology simulator—a survey study
Source: Adv Simul (Lond). 2024 Feb 14;9:9. doi: 10.1186/s41077-024-00280-9 (PMC10865533; doi:10.1186/s41077-024-00280-9)

**Appendix 1**

**
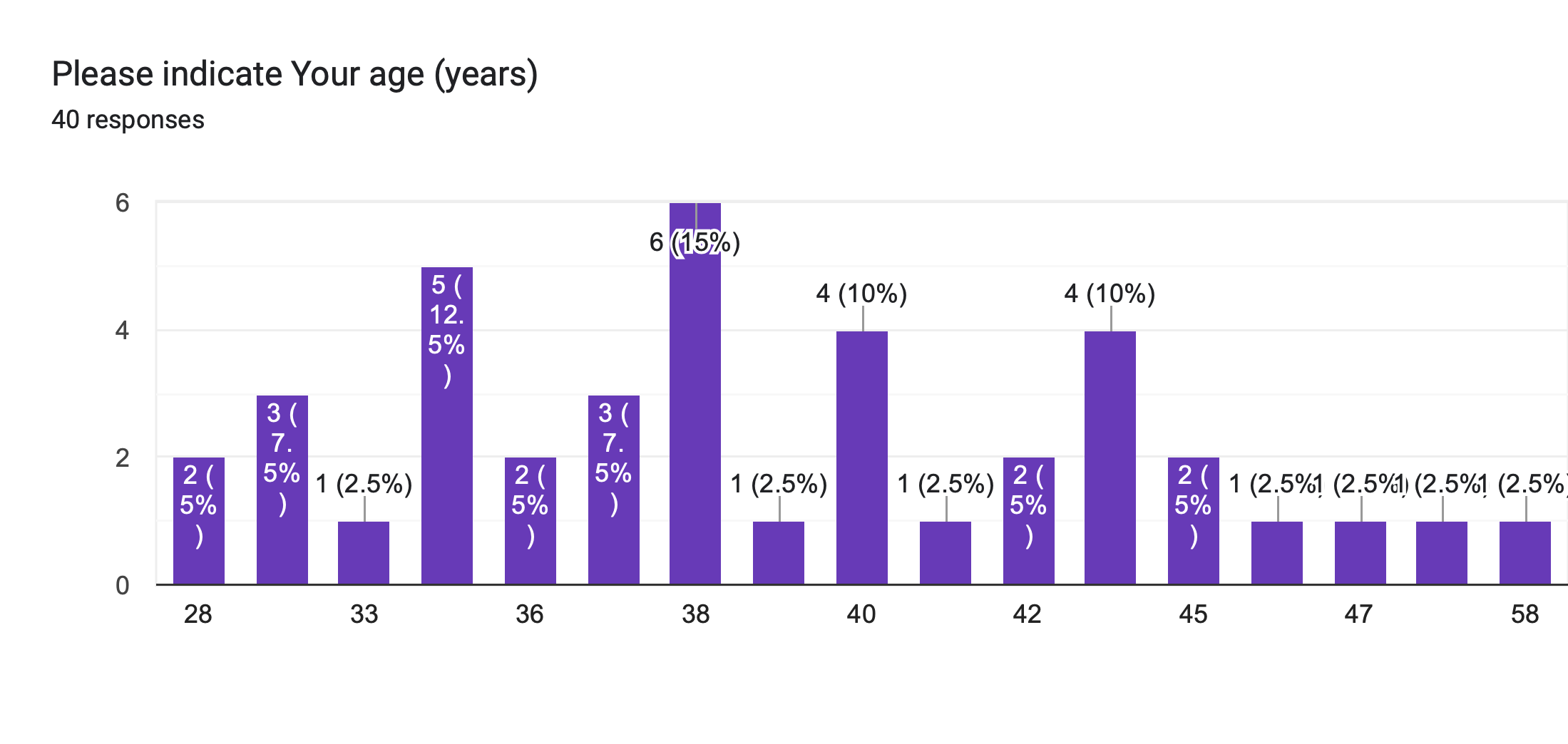
**

**
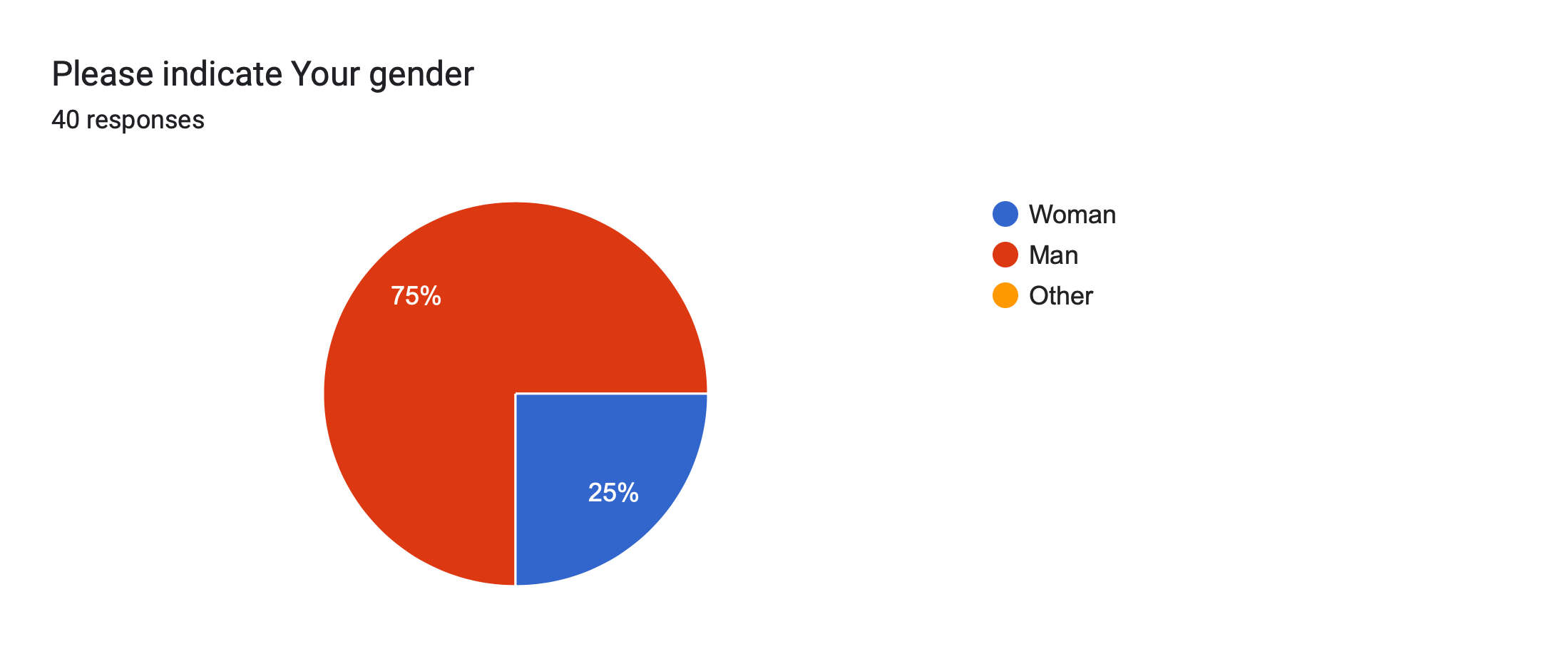
**

**
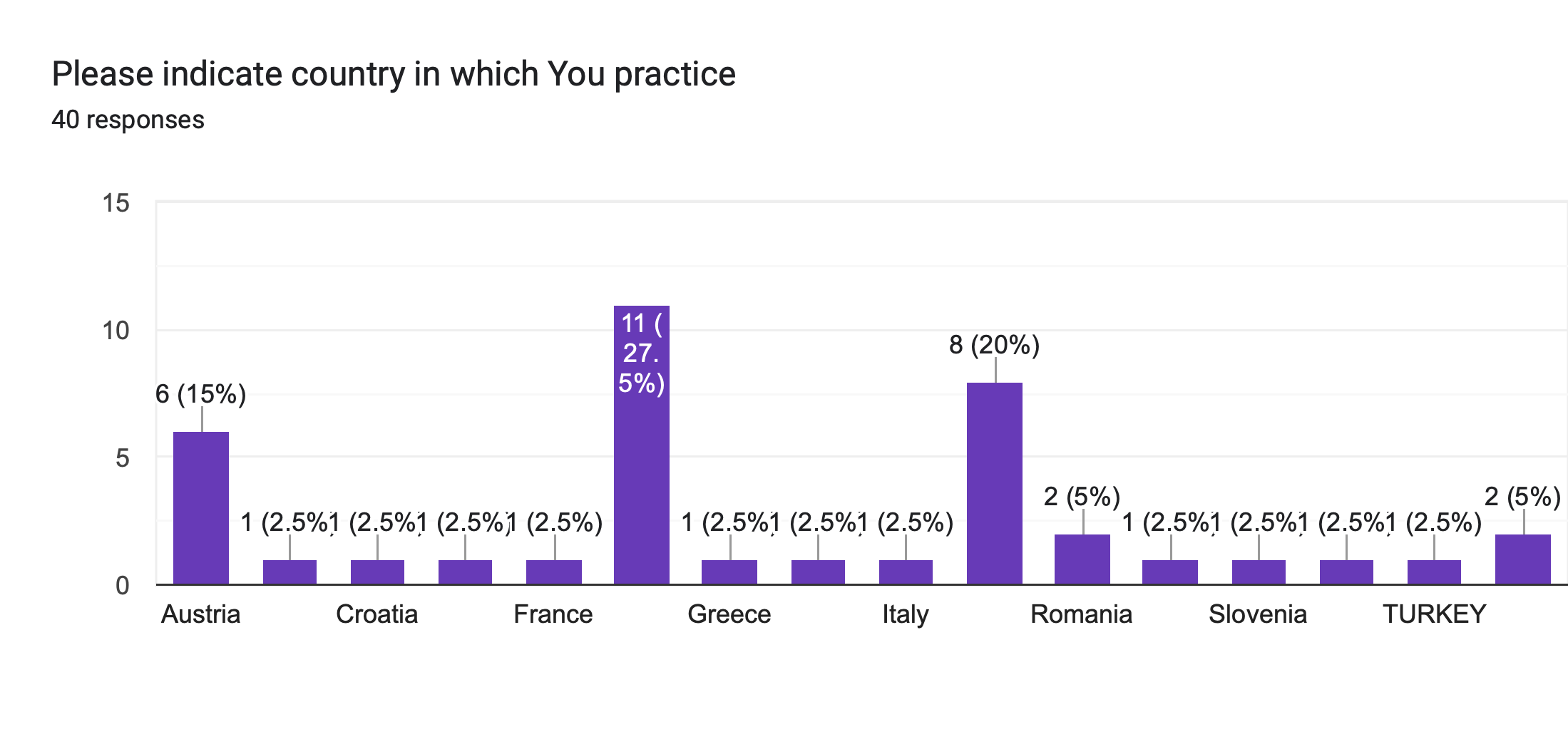
**

**
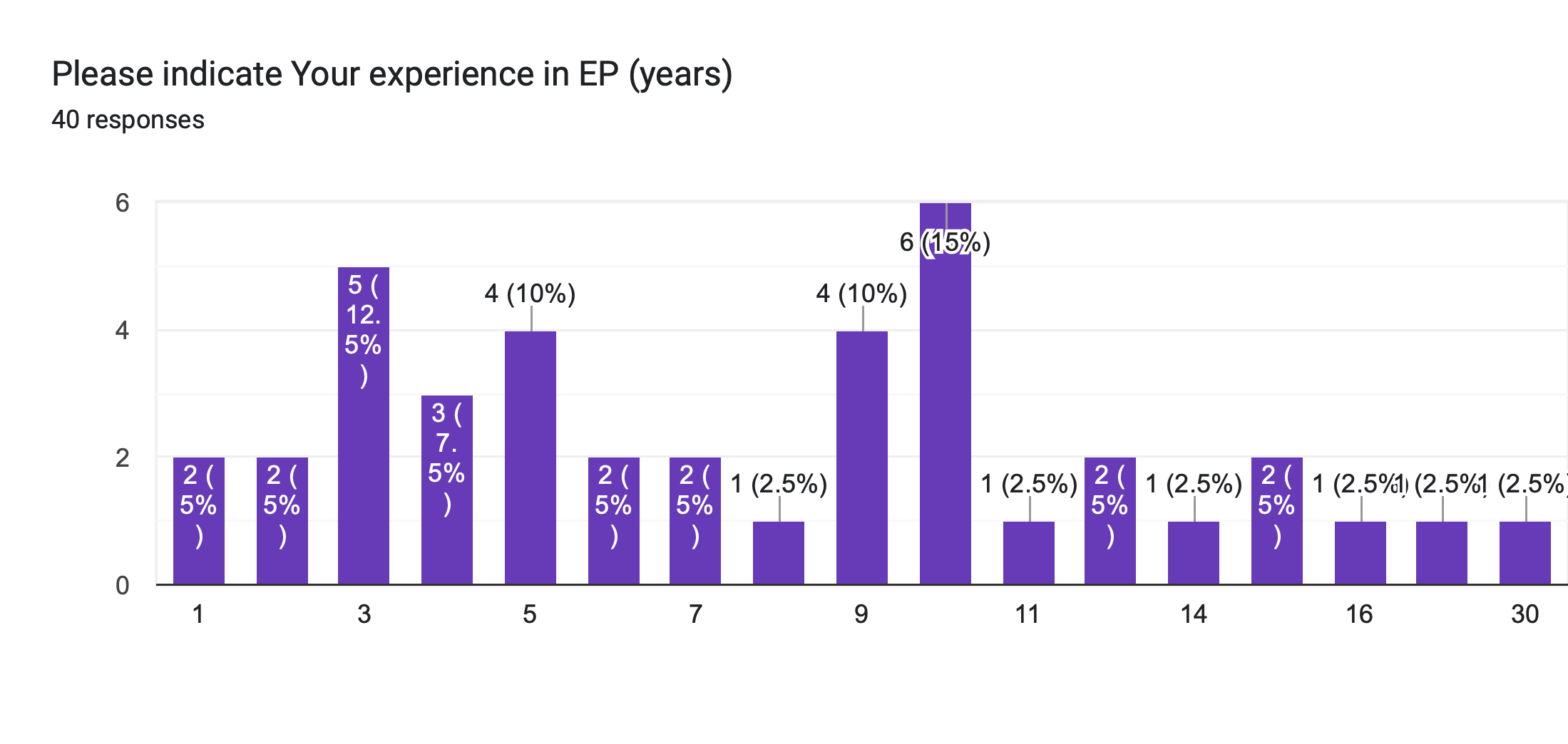
**

**
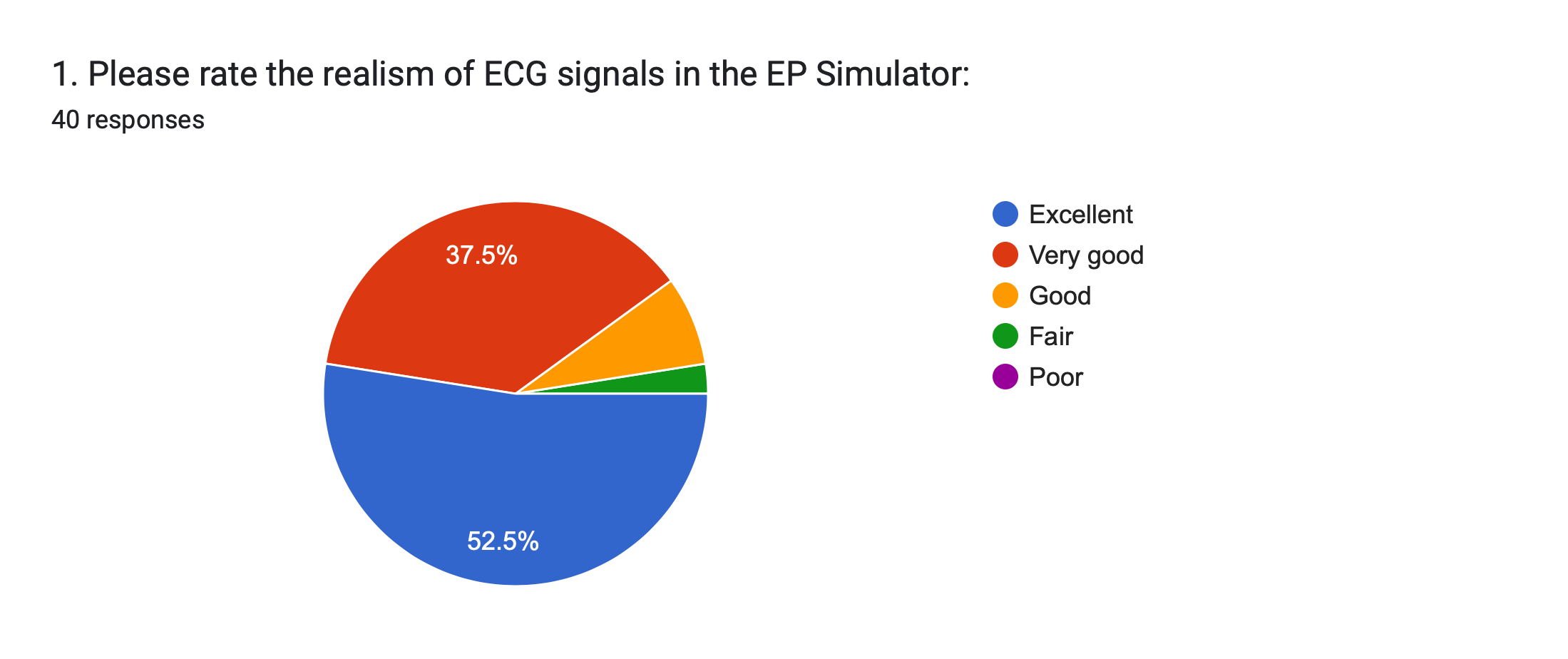
**

**
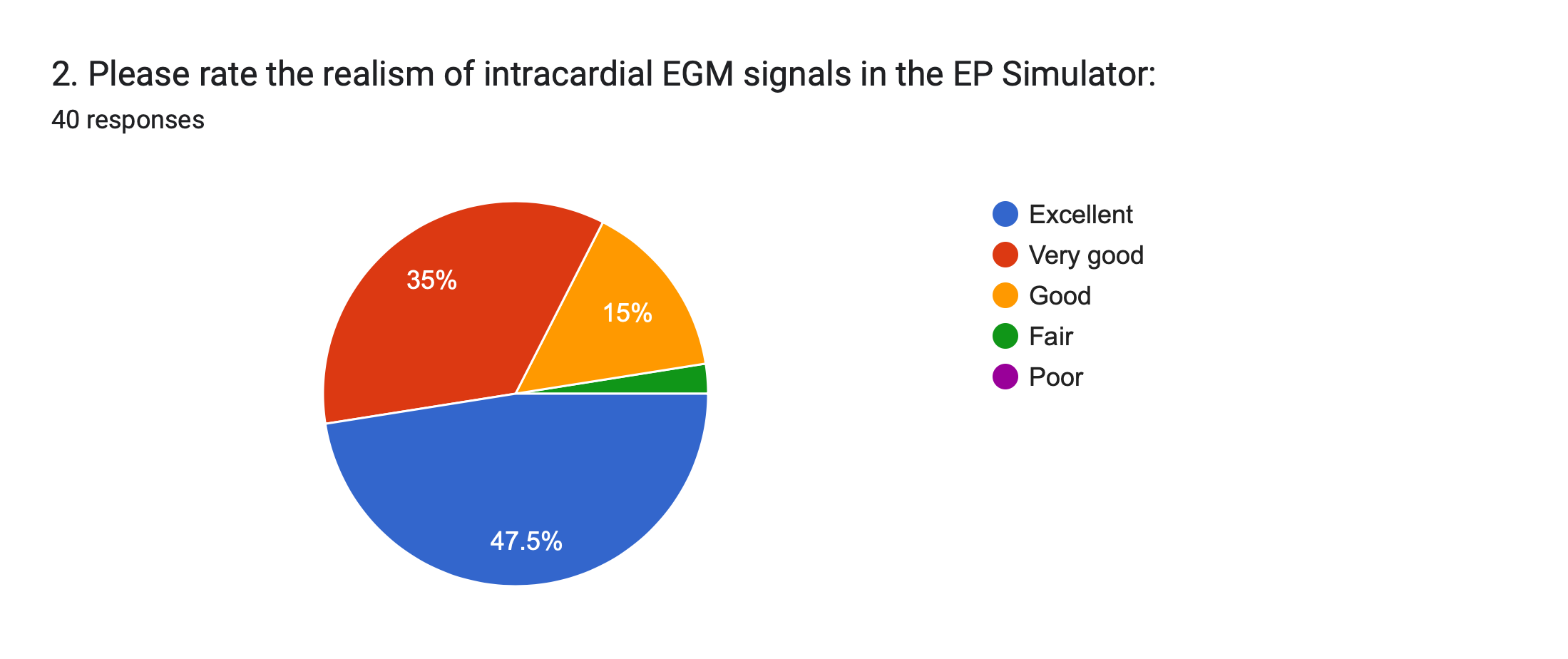
**

**
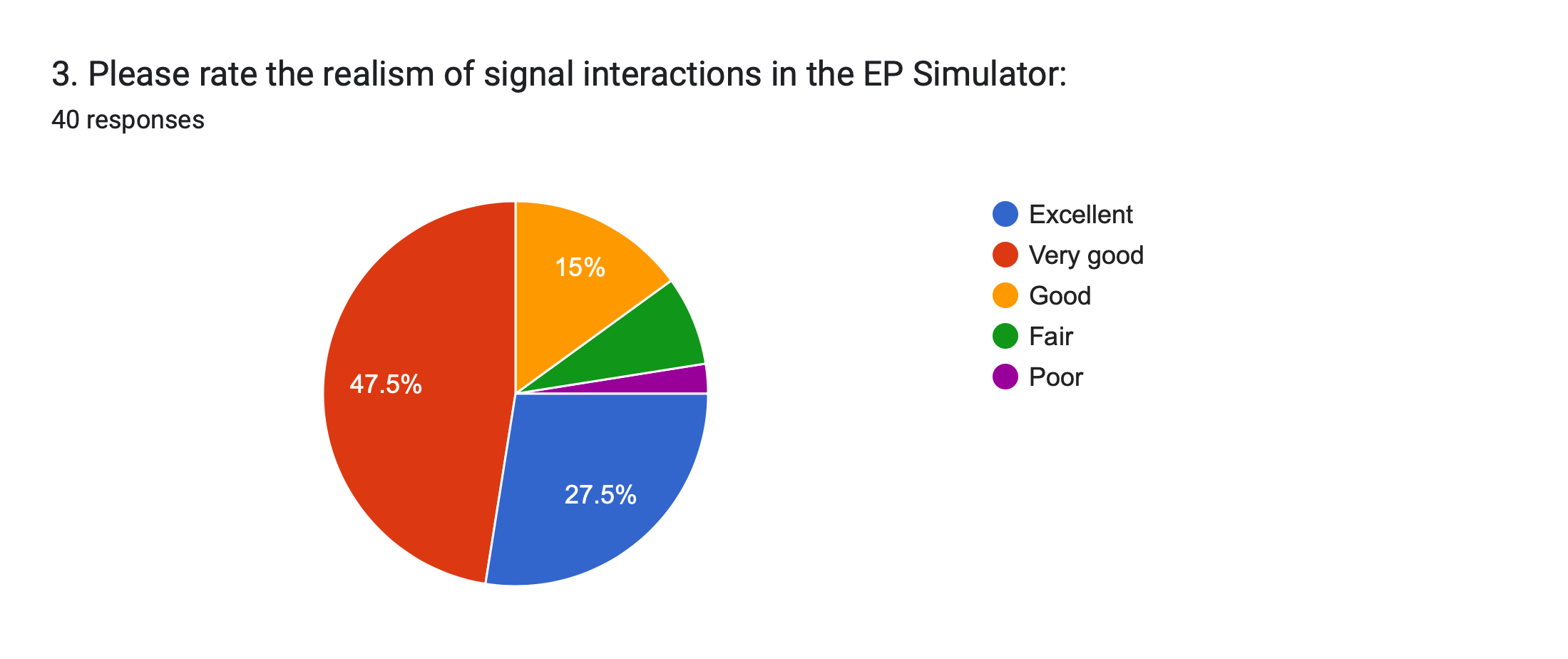
**

**
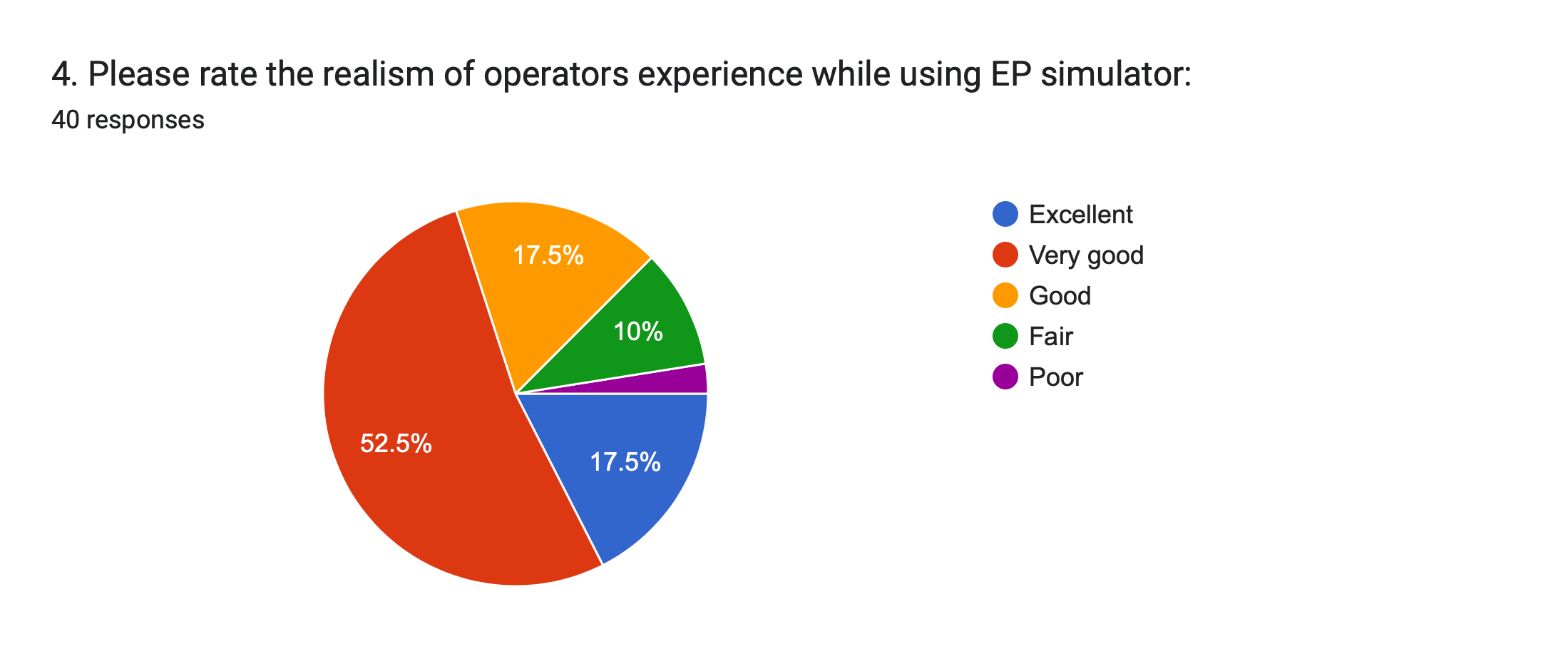
**

**
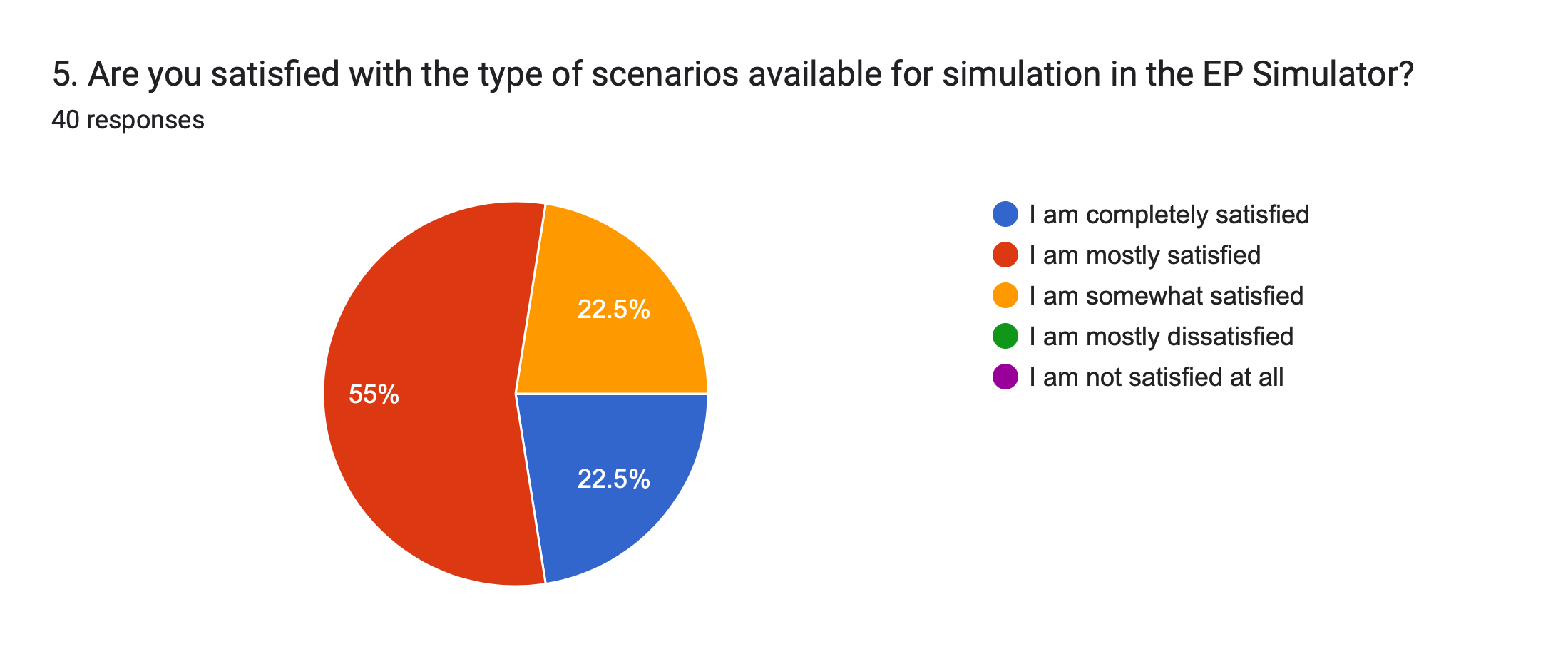
**

**
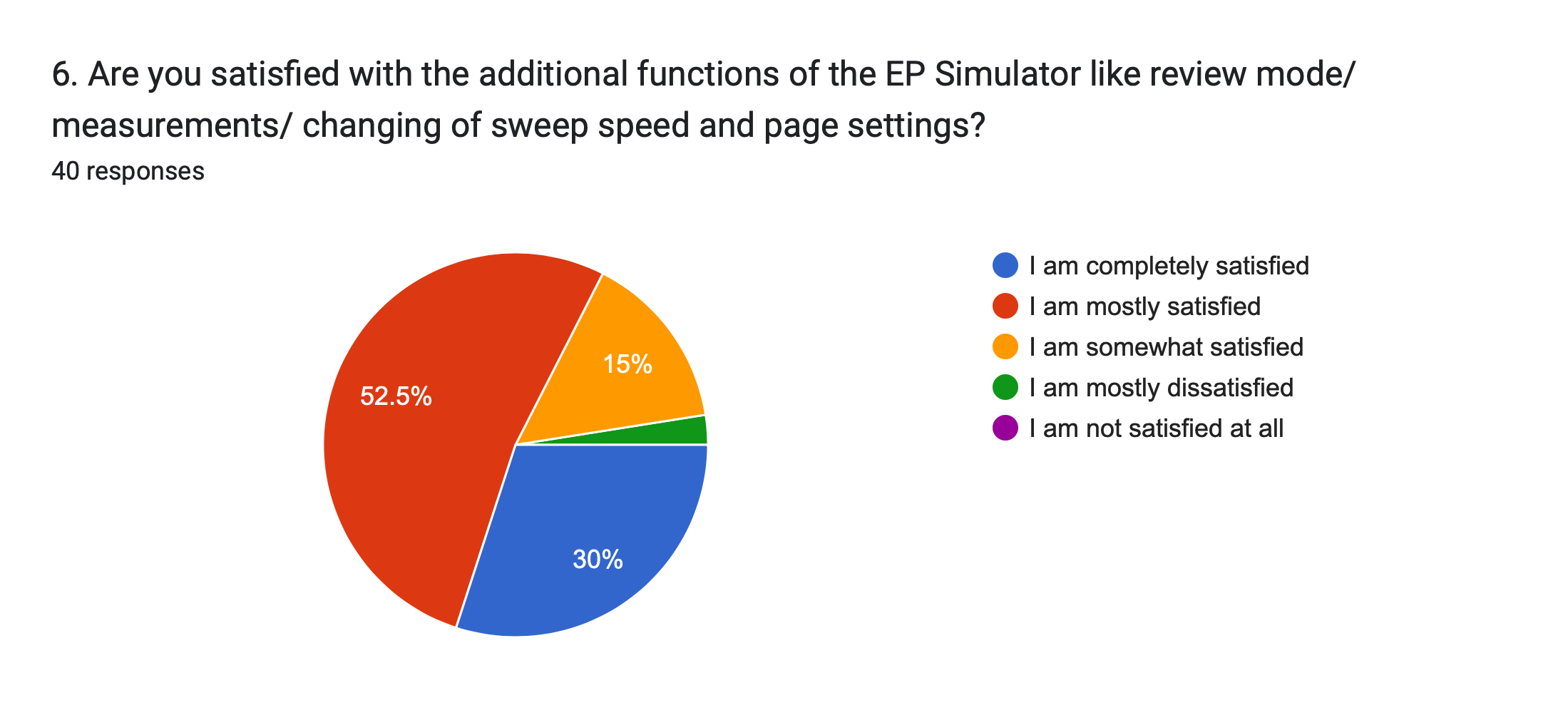
**

**
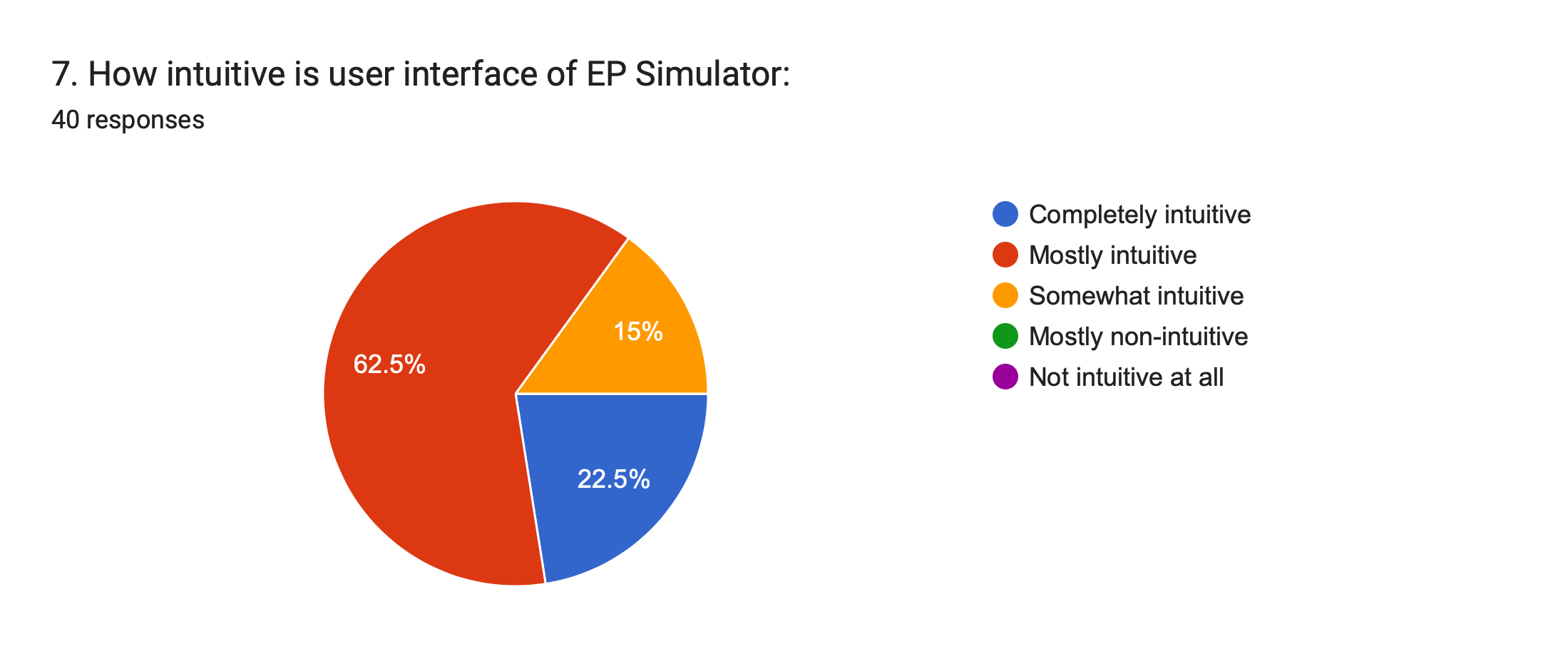
**

**
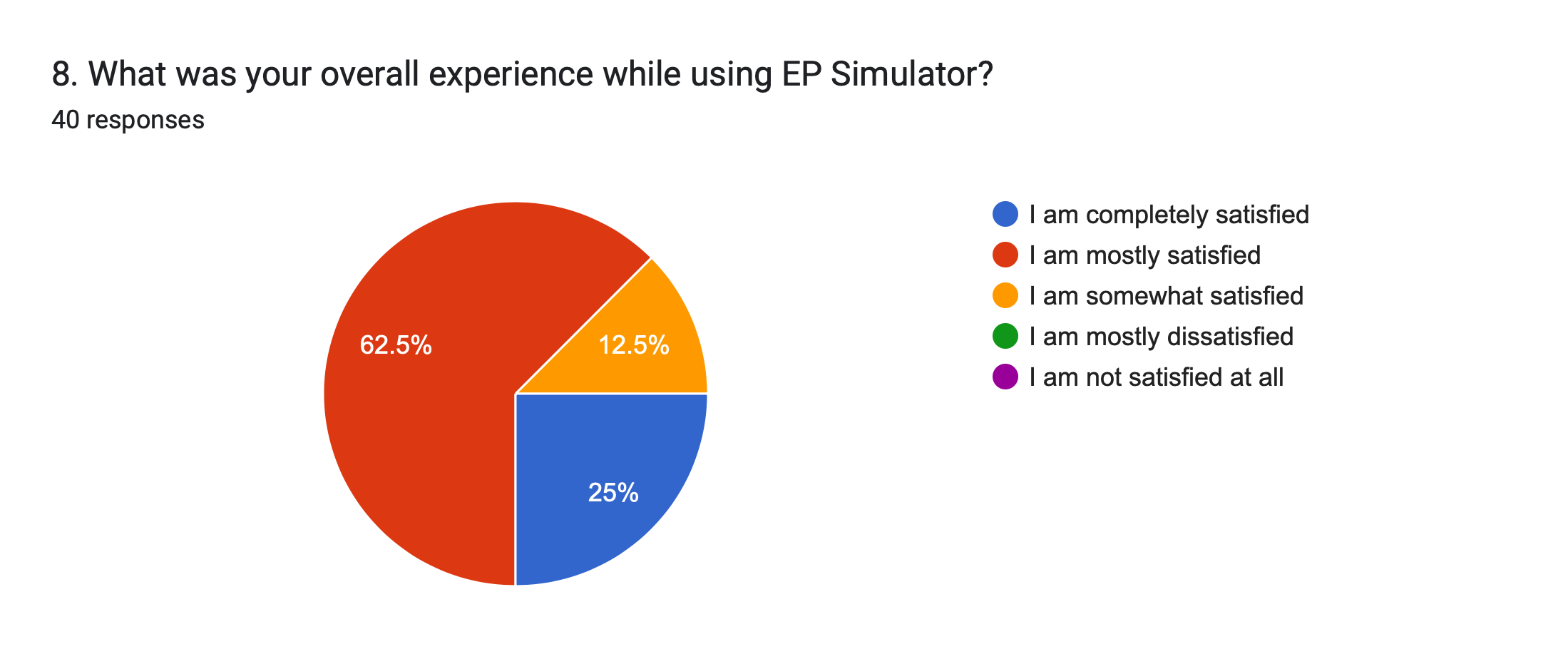
**

**
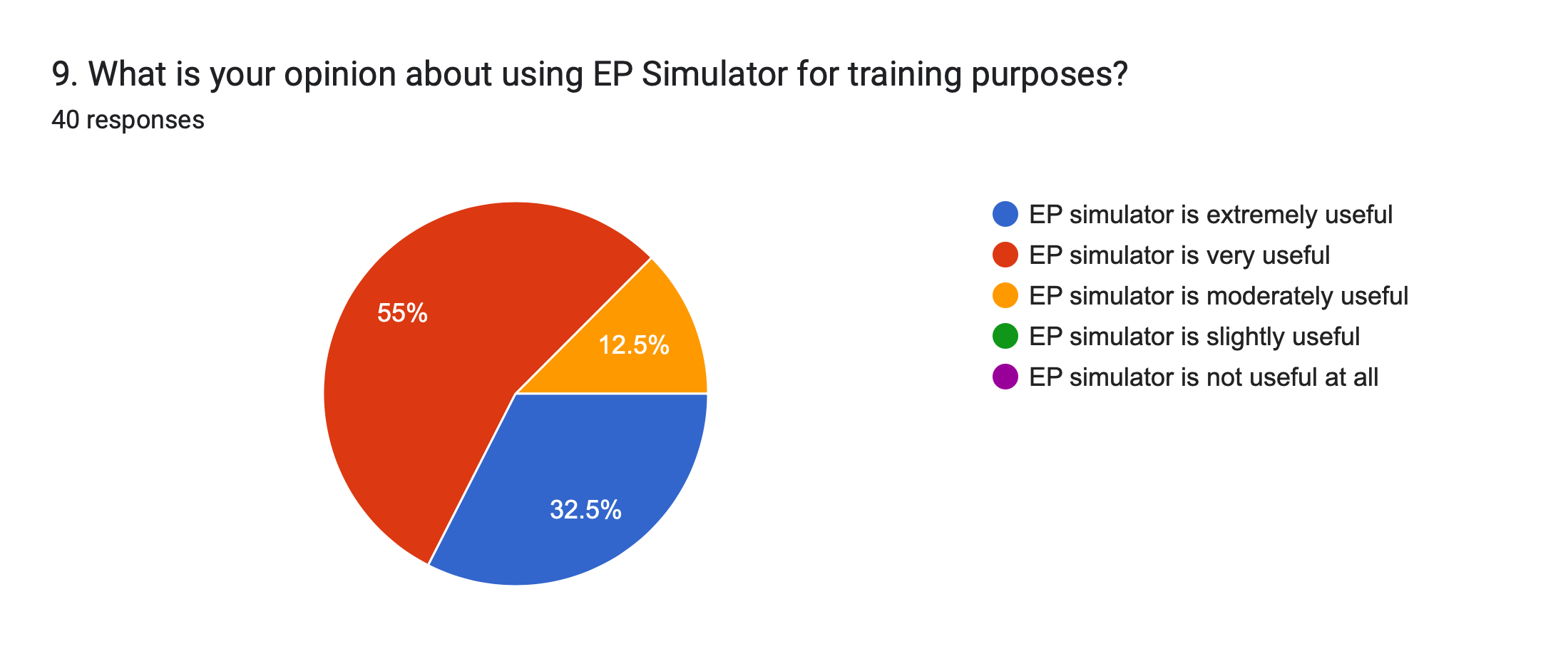
**

**
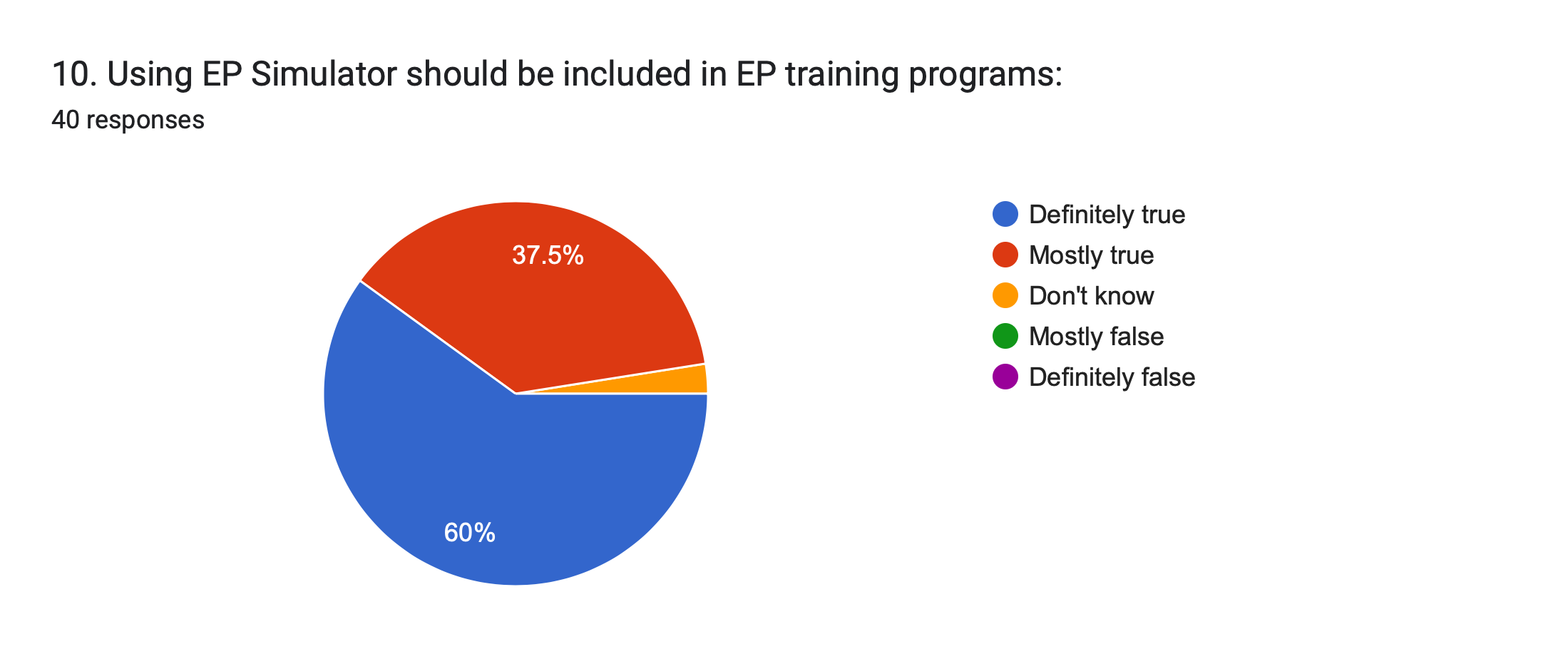
**

**
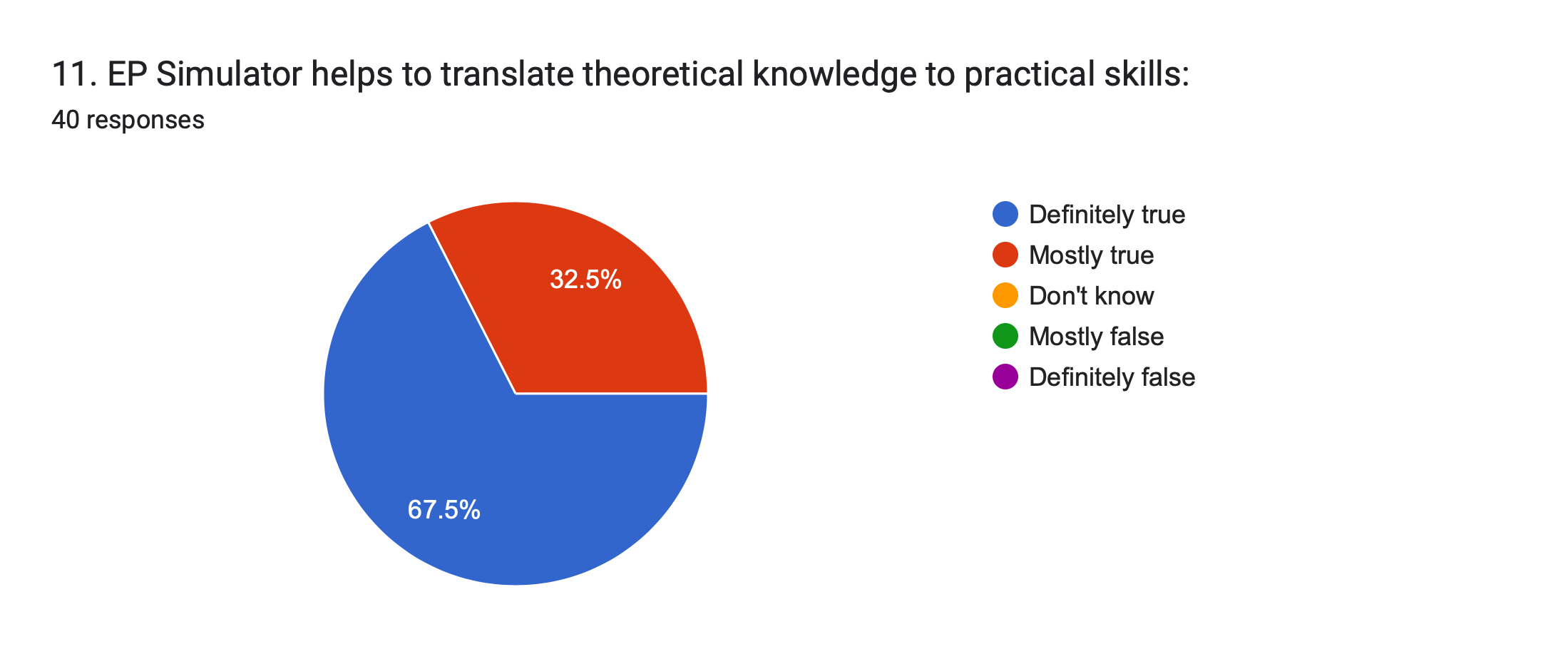
**

**
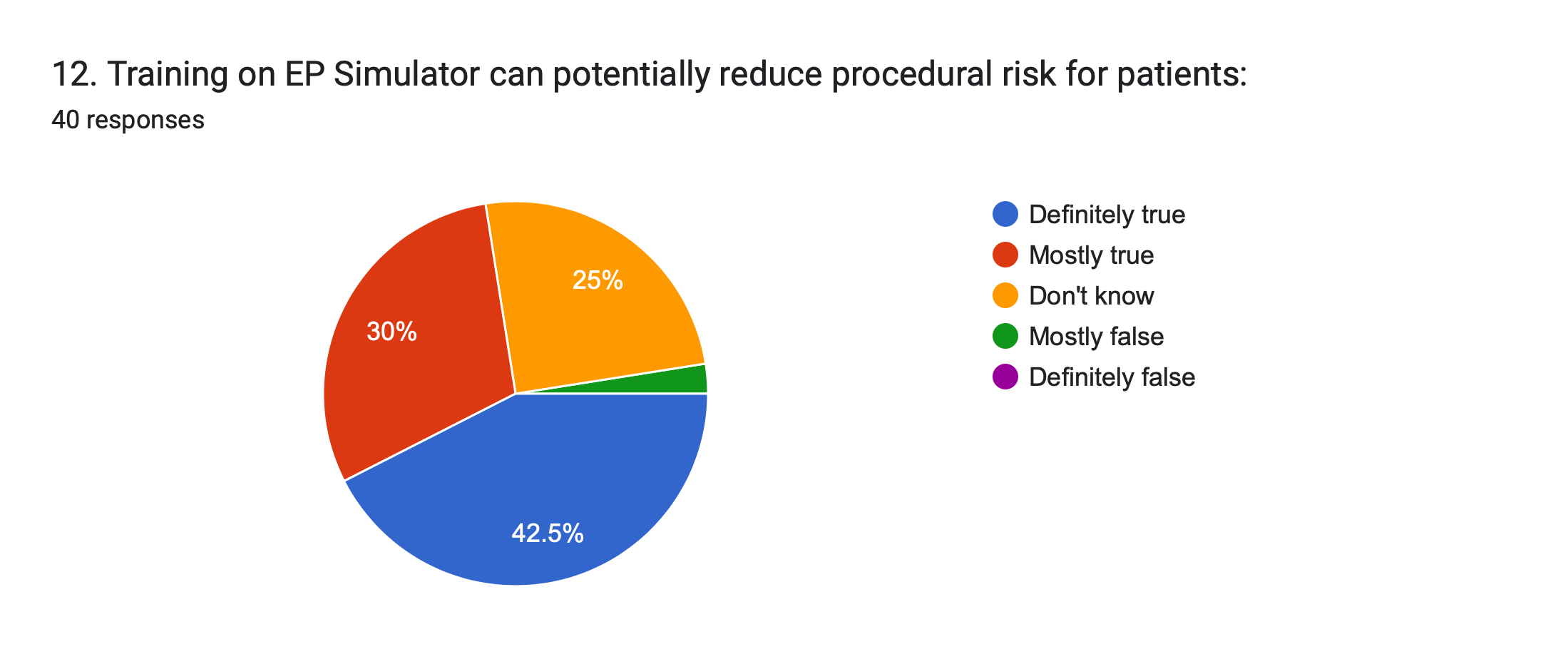
**

**
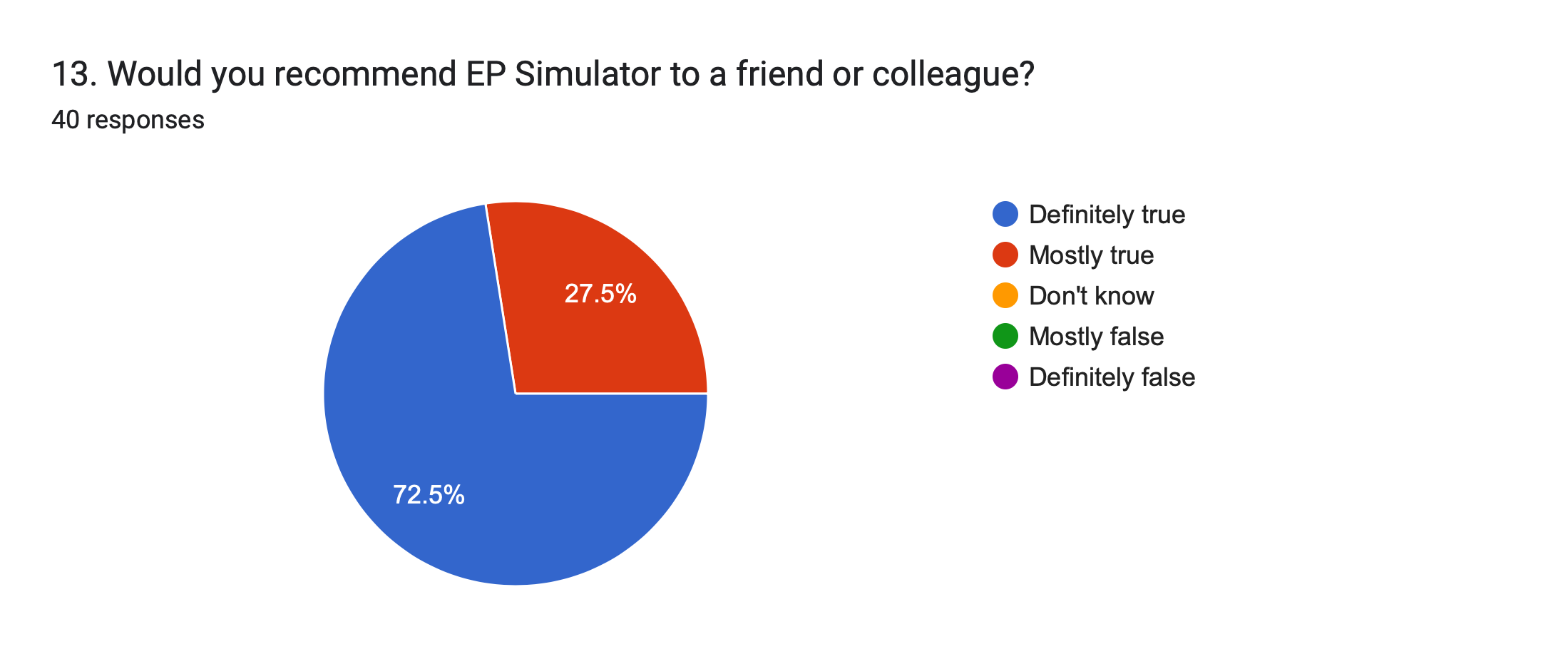
**

Supplement: Supplementary file 1 — Additional file 1. [file 41077_2024_280_MOESM1_ESM.docx]
